# Supplementary material for: Comparison of calling pipelines for whole genome sequencing: an empirical study demonstrating the importance of mapping and alignment
Source: Sci Rep. 2022 Dec 13;12:21502. doi: 10.1038/s41598-022-26181-3 (PMC9748128; doi:10.1038/s41598-022-26181-3)
Supplement: Supplementary file 1 — Supplementary Information. [file 41598_2022_26181_MOESM1_ESM.pdf]

Supplementary material to

Comparison of calling pipelines for whole genome sequencing: an empirical study demonstrating the importance of mapping and alignment

By Raphael O. Betschart, Alexandre Thiéry, Domingo Aguilera-Garcia, Martin Zoche, Holger Moch, Raphael Twerenbold, Tanja Zeller, Stefan Blankenberg, and Andreas Ziegler

Supplementary Figures

Figure S1: Runtime comparisons. Left: between the DRAGEN and GATK pipeline for mapping & alignment. Right: between DRAGEN, DeepVariant (DV), GATK mapping & alignment plus GATK Haplotypecaller in the DRAGEN mode (GATK / DRAGEN), and GATK mapping & alignment plus GATK Haplotypecaller not in the DRAGEN mode (GATK / GATK).

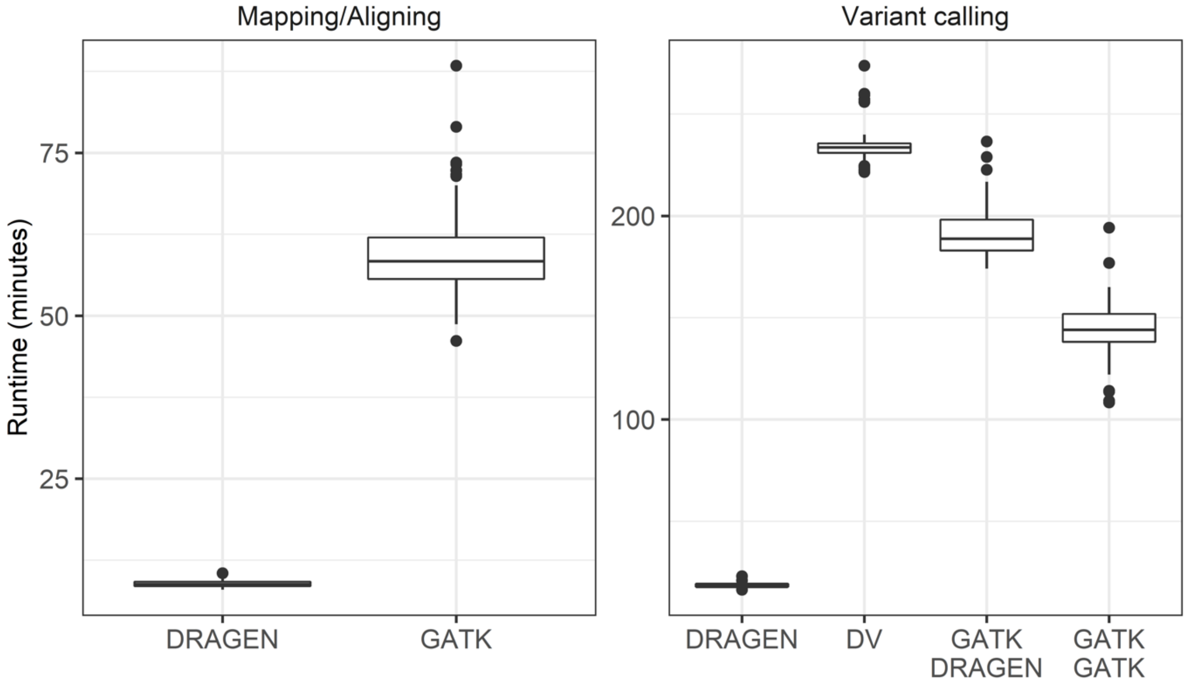

Figure S2: Performance evaluation of all 6 pipelines combination for single nucleotide variations (SNVs) and Indels, based on all autosomes of the genome in a bottle sample HG003. D: DRAGEN; DV: DeepVariant; G: GATK. First row in legend on x-axis displays approach used for mapping & alignment. Second row in legend on x-axis displays variant caller used. In detail: D/D: DRAGEN for mapping & alignment and variant calling. D/DV: DRAGEN for mapping & alignment, DeepVariant for variant calling. D/G: DRAGEN for mapping & alignment, GATK with Haplotypecaller not in the DRAGEN mode for variant calling. G/D: GATK for mapping & alignment, GATK with Haplotypecaller in the DRAGEN mode for variant calling. G/DV: GATK for mapping & alignment, DeepVariant for variant calling. G/G: GATK for mapping & alignment, GATK without Haplotypecaller in the DRAGEN mode for variant calling.

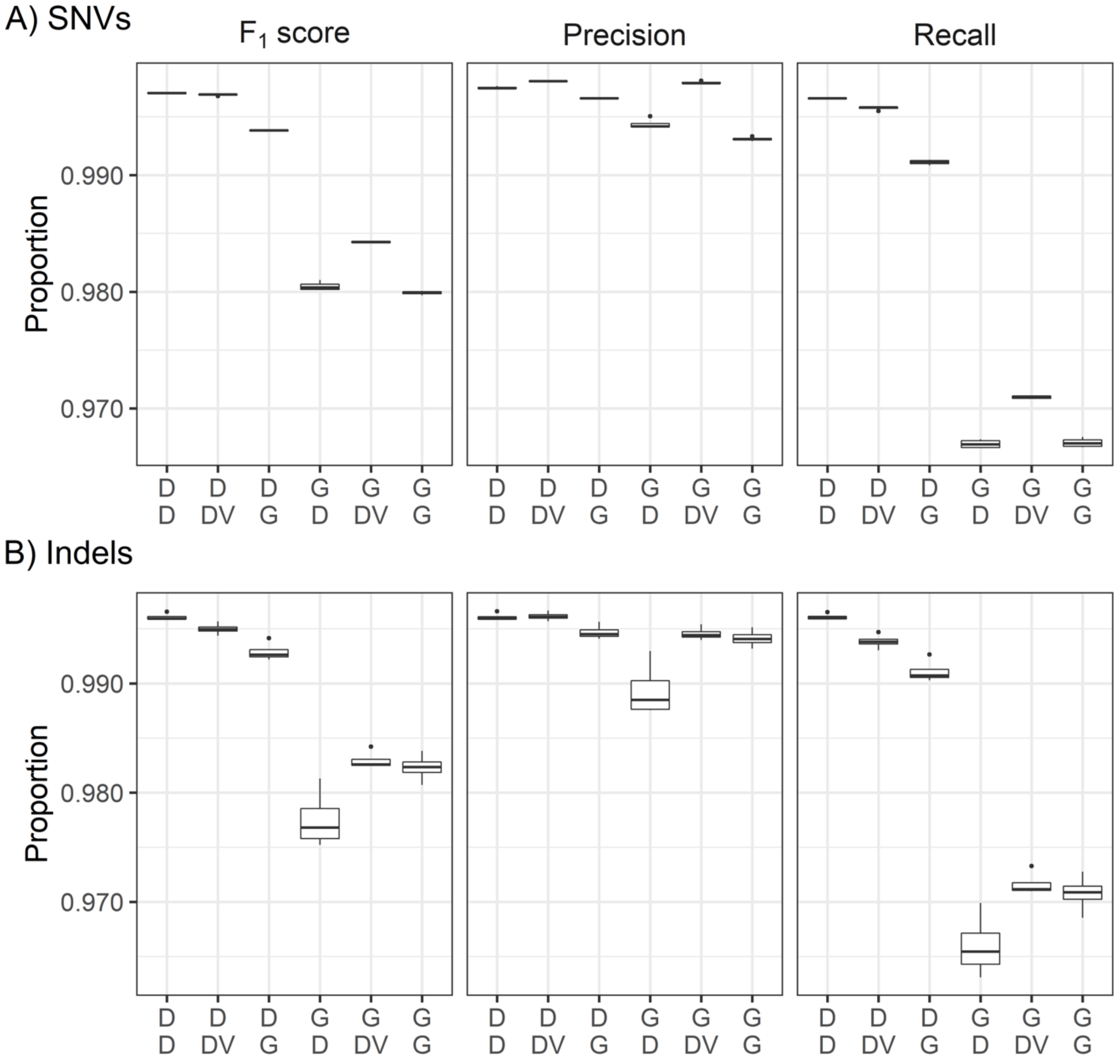

Figure S3: Percentages of F_1 score, precision, and recall for insertions in complex regions (upper part A) and insertions in simple regions (lower part B) for short (1-5 bp), medium (6-15 bp), and long (> 15 bp) insertions for the 6 pipeline combinations, based on chromosomes 20 to 22 of the genome in a bottle sample HG002. Labels on the x-axis are defined in detail in Supplementary Figure S3.

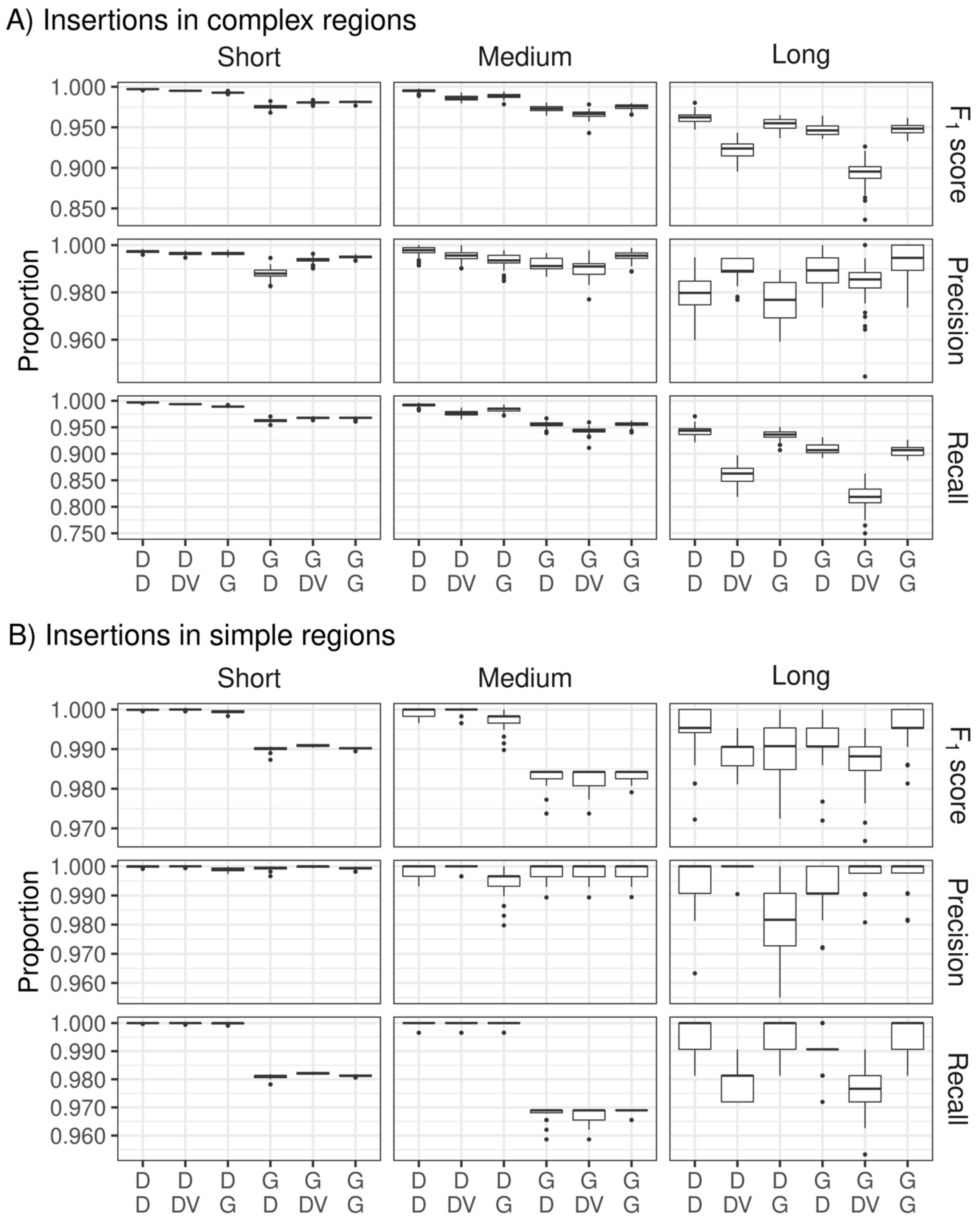

Figure S4: Percentages of F_1 score, precision, and recall for deletions in complex regions (upper part A) and deletions in simple regions (lower part B) for short (1-5 bp), medium (6-15 bp), and long (> 15 bp) deletions for the 6 pipeline combinations, based on chromosomes 20 to 22 of the genome in a bottle sample HG002. Labels on the x-axis are defined in detail in Supplementary Figure S3.

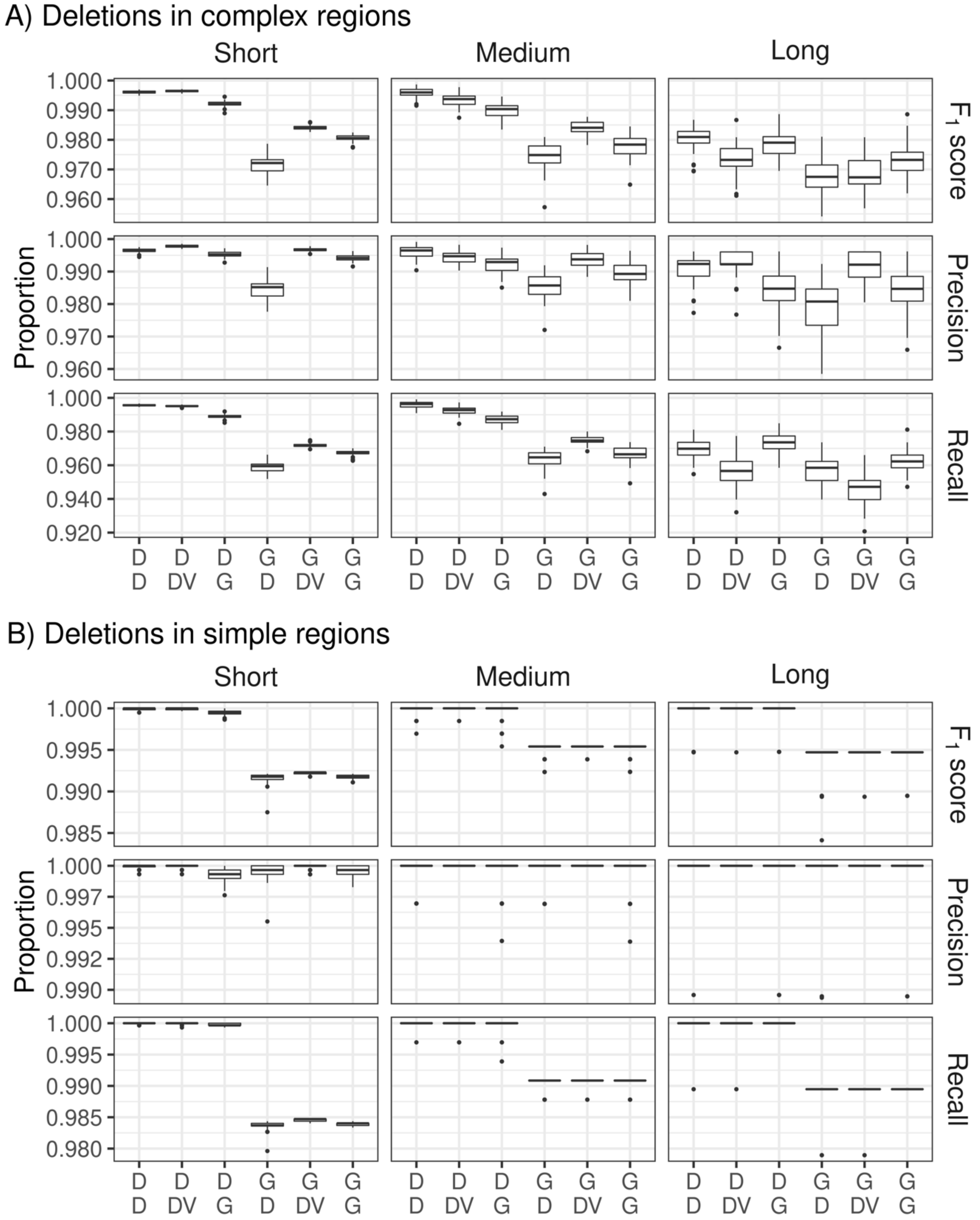

Figure S5: Performance evaluation of three pipelines for varying target sensitivities using the genome in a bottle sample HG002. Red: GATK Haplotypecaller (in DRAGEN mode, based on the bam file obtained from GATK with BWA-MEM2 mapping & alignment). Blue: GATK Haplotypecaller (not in DRAGEN mode, based on the bam file obtained from DRAGEN). Green: GATK Haplotypecaller (not in DRAGEN mode, based on the bam file obtained from GATK with BWA-MEM2 mapping & alignment). SNV: single nucleotide variation.

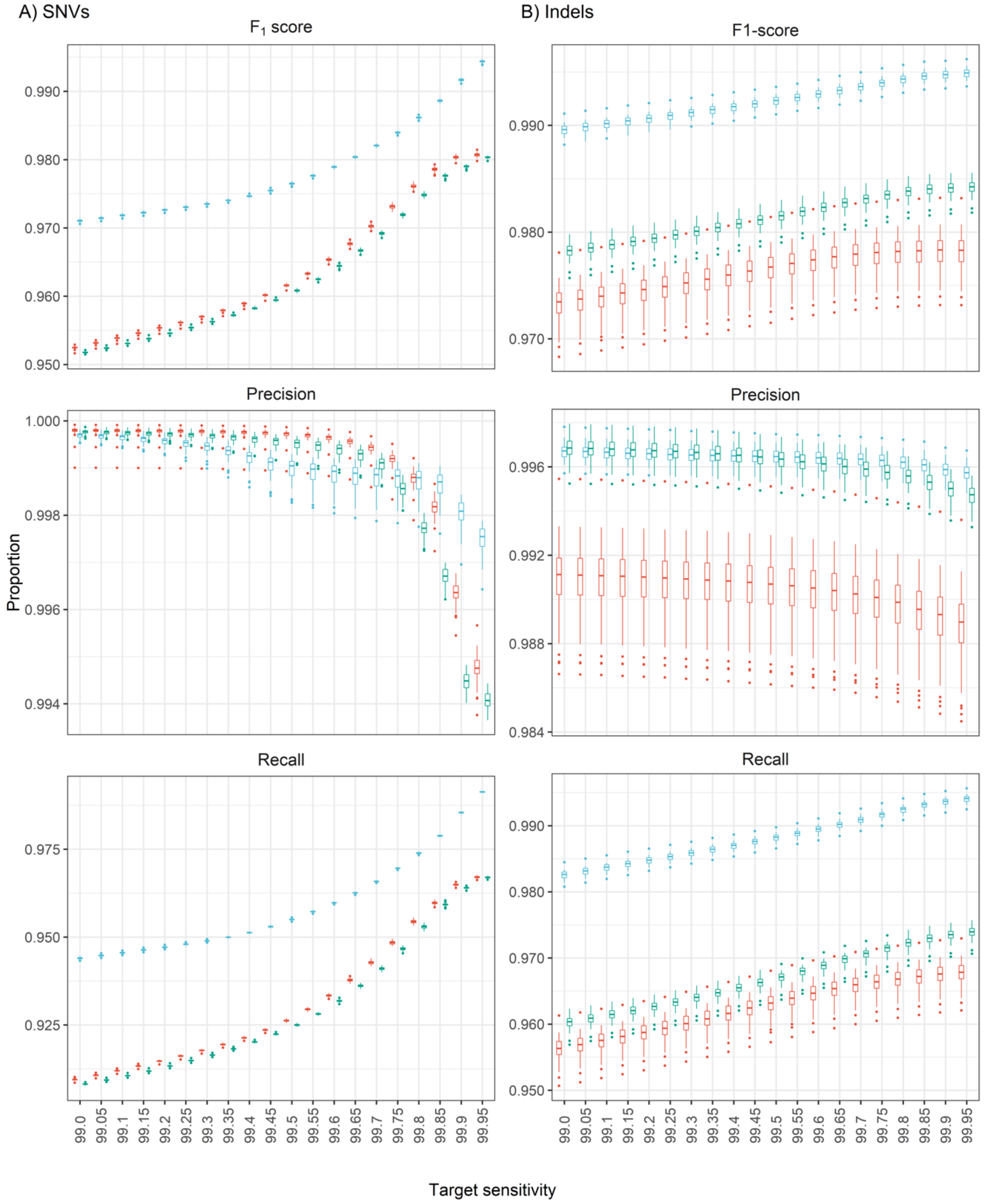

Table S1: Summary table of mean differences and 95% confidence intervals between the runtime of all 15 pipeline combinations.

Pipeline 1	Pipeline 2	Mean difference	CI low	CI high
D-D	D-DV	-220	-222	-218
D-D	D-G	-134	-138	-129
D-D	G-D	-347	-356	-337
D-D	G-DV	-385	-394	-376
D-D	G-G	-291	-300	-281
D-DV	D-G	86	82	90
D-DV	G-D	-127	-136	-117
D-DV	G-DV	-165	-174	-156
D-DV	G-G	-70	-80	-61
D-G	G-D	-213	-222	-203
D-G	G-DV	-251	-261	-242
D-G	G-G	-157	-166	-148
G-D	G-DV	-38	-43	-34
G-D	G-G	56	54	58
G-DV	G-G	95	90	100

Table S2: Summary table of mean differences and 95% confidence intervals of the performance for F_1 score, recall and precision for SNVs and Indels in simple, complex, coding, and non-coding and all regions for chromosomes 20 to 22 of HG002.

Type	Region	Metric	Pipeline 1	Pipeline 2	Mean difference	CI low	CI high
Indel	All regions	Precision	D-D	D-DV	-0.000690655882	-0.0009643992908	-0.000416912474
Indel	All regions	Precision	D-D	D-G	0.002252808824	0.0019625243723	0.002543093275
Indel	All regions	Precision	D-D	G-D	0.004013229412	0.0035957459520	0.004430712872
Indel	All regions	Precision	D-D	G-DV	0.000710238235	0.0004093939092	0.001011082561
Indel	All regions	Precision	D-D	G-G	0.000862525000	0.0005085112640	0.001216538736
Indel	All regions	Precision	D-DV	D-G	0.002943464706	0.0025266007336	0.003360328678
Indel	All regions	Precision	D-DV	G-D	0.004703885294	0.0043473942019	0.005060376386
Indel	All regions	Precision	D-DV	G-DV	0.001400894118	0.0011946264648	0.001607161771
Indel	All regions	Precision	D-DV	G-G	0.001553180882	0.0013072836074	0.001799078157
Indel	All regions	Precision	D-G	G-D	0.001760420588	0.0012652287796	0.002255612397
Indel	All regions	Precision	D-G	G-DV	-0.001542570588	-0.0019467069815	-0.001138434195
Indel	All regions	Precision	D-G	G-G	-0.001390283824	-0.0018368468431	-0.000943720804
Indel	All regions	Precision	G-DV	G-G	0.000152286765	-0.0001830383438	0.000487611873

Type	Region	Metric	Pipeline 1	Pipeline 2	Mean difference	CI low	CI high
Indel	All regions	Recall	D-D	D-DV	0.008774636765	0.0074499026287	0.010099370901
Indel	All regions	Recall	D-D	D-G	0.003233710294	0.0029278131103	0.003539607478
Indel	All regions	Recall	D-D	G-D	0.019208885294	0.0181387143172	0.020279056271
Indel	All regions	Recall	D-D	G-DV	0.024468611765	0.0225395681580	0.026397655371
Indel	All regions	Recall	D-D	G-G	0.016968577941	0.0159821861907	0.017954969692
Indel	All regions	Recall	D-DV	D-G	-0.005540926471	-0.0068302393304	-0.004251613611
Indel	All regions	Recall	D-DV	G-D	0.010434248529	0.0089207267673	0.011947770292
Indel	All regions	Recall	D-DV	G-DV	0.015693975000	0.0147614351782	0.016626514822
Indel	All regions	Recall	D-DV	G-G	0.008193941176	0.0067889611576	0.009598921195
Indel	All regions	Recall	D-G	G-D	0.015975175000	0.0151010919445	0.016849258055
Indel	All regions	Recall	D-G	G-DV	0.021234901471	0.0194227590430	0.023047043898
Indel	All regions	Recall	D-G	G-G	0.013734867647	0.0129620076198	0.014507727674
Indel	All regions	Recall	G-D	G-DV	0.005259726471	0.0036747244094	0.006844728532
Indel	All regions	Recall	G-D	G-G	-0.002240307353	-0.0024887910494	-0.001991823656

Type	Region	Metric	Pipeline 1	Pipeline 2	Mean difference	CI low	CI high
Indel	All regions	Recall	G-DV	G-G	-0.007500033824	-0.0090134926434	-0.005986575004
Indel	All regions	F1-score	D-D	D-DV	0.006094848739	0.0052388150430	0.006950882436
Indel	All regions	F1-score	D-D	D-G	0.003914892857	0.0036356668309	0.004194118883
Indel	All regions	F1-score	D-D	G-D	0.016795546218	0.0163320229564	0.017259069481
Indel	All regions	F1-score	D-D	G-DV	0.018666602941	0.0175074765185	0.019825729364
Indel	All regions	F1-score	D-D	G-G	0.012972798319	0.0125471613147	0.013398435324
Indel	All regions	F1-score	D-DV	D-G	-0.002179955882	-0.0029794073372	-0.001380504427
Indel	All regions	F1-score	D-DV	G-D	0.010700697479	0.0095614374196	0.011839957538
Indel	All regions	F1-score	D-DV	G-DV	0.012571754202	0.0120680707401	0.013075437663
Indel	All regions	F1-score	D-DV	G-G	0.006877949580	0.0058279764663	0.007927922693
Indel	All regions	F1-score	D-G	G-D	0.012880653361	0.0123813302231	0.013379976500
Indel	All regions	F1-score	D-G	G-DV	0.014751710084	0.0136688064546	0.015834613713
Indel	All regions	F1-score	D-G	G-G	0.009057905462	0.0086500447700	0.009465766154
Indel	All regions	F1-score	G-D	G-DV	0.001871056723	0.0005037095782	0.003238403867

Type	Region	Metric	Pipeline 1	Pipeline 2	Mean difference	CI low	CI high
Indel	All regions	F1-score	G-D	G-G	-0.003822747899	-0.0041104780635	-0.003535017735
Indel	All regions	F1-score	G-DV	G-G	-0.005693804622	-0.0069584617027	-0.004429147541
Indel	Complex regions	Precision	D-D	D-DV	-0.000728613235	-0.0010802008097	-0.000377025661
Indel	Complex regions	Precision	D-D	D-G	0.002305104412	0.0019707834268	0.002639425397
Indel	Complex regions	Precision	D-D	G-D	0.005108085294	0.0045302464963	0.005685924092
Indel	Complex regions	Precision	D-D	G-DV	0.001090097059	0.0006808887271	0.001499305391
Indel	Complex regions	Precision	D-D	G-G	0.001006380882	0.0005259458853	0.001486815879
Indel	Complex regions	Precision	D-DV	D-G	0.003033717647	0.0025698561025	0.003497579192
Indel	Complex regions	Precision	D-DV	G-D	0.005836698529	0.0053516764273	0.006321720631
Indel	Complex regions	Precision	D-DV	G-DV	0.001818710294	0.0015344893771	0.002102931211
Indel	Complex regions	Precision	D-DV	G-G	0.001734994118	0.0013902220990	0.002079766136
Indel	Complex regions	Precision	D-G	G-D	0.002802980882	0.0021833698896	0.003422591875
Indel	Complex regions	Precision	D-G	G-DV	-0.001215007353	-0.0016901510576	-0.000739863648
Indel	Complex regions	Precision	D-G	G-G	-0.001298723529	-0.0018191311449	-0.000778315914

Type	Region	Metric	Pipeline 1	Pipeline 2	Mean difference	CI low	CI high
Indel	Complex regions	Precision	G-D	G-DV	-0.004017988235	-0.0045922955377	-0.003443680933
Indel	Complex regions	Precision	G-D	G-G	-0.004101704412	-0.0044658204265	-0.003737588397
Indel	Complex regions	Precision	G-DV	G-G	-0.000083716176	-0.0005561120034	0.000388679650
Indel	Complex regions	Recall	D-D	D-DV	0.012006510294	0.0101589686952	0.013854051893
Indel	Complex regions	Recall	D-D	D-G	0.004434704412	0.0040161819637	0.004853226860
Indel	Complex regions	Recall	D-D	G-D	0.022291333824	0.0210387095875	0.023543958060
Indel	Complex regions	Recall	D-D	G-DV	0.029913170588	0.0272324364741	0.032593904702
Indel	Complex regions	Recall	D-D	G-G	0.019618007353	0.0184446984565	0.020791316249
Indel	Complex regions	Recall	D-DV	D-G	-0.007571805882	-0.0093475772594	-0.005796034505
Indel	Complex regions	Recall	D-DV	G-D	0.010284823529	0.0083957306257	0.012173916433
Indel	Complex regions	Recall	D-DV	G-DV	0.017906660294	0.0167620193352	0.019051301253
Indel	Complex regions	Recall	D-DV	G-G	0.007611497059	0.0059121434600	0.009310850658
Indel	Complex regions	Recall	D-G	G-D	0.017856629412	0.0168734537920	0.018839805031
Indel	Complex regions	Recall	D-G	G-DV	0.025478466176	0.0229575945531	0.027999337800

Type	Region	Metric	Pipeline 1	Pipeline 2	Mean difference	CI low	CI high
Indel	Complex regions	Recall	D-G	G-G	0.015183302941	0.0143072226837	0.016059383199
Indel	Complex regions	Recall	G-D	G-DV	0.007621836765	0.0053884645092	0.009855209020
Indel	Complex regions	Recall	G-D	G-G	-0.002673326471	-0.0030288242688	-0.002317828672
Indel	Complex regions	Recall	G-DV	G-G	-0.010295163235	-0.0123735682222	-0.008216758248
Indel	Complex regions	F1-score	D-D	D-DV	0.008698913866	0.0074371439759	0.009960683755
Indel	Complex regions	F1-score	D-D	D-G	0.004824123950	0.0044795472005	0.005168700699
Indel	Complex regions	F1-score	D-D	G-D	0.019904638655	0.0193484059792	0.020460871332
Indel	Complex regions	F1-score	D-D	G-DV	0.023428159664	0.0216270708862	0.025229248442
Indel	Complex regions	F1-score	D-D	G-G	0.015127084034	0.0146379329445	0.015616235123
Indel	Complex regions	F1-score	D-DV	D-G	-0.003874789916	-0.0050873340159	-0.002662245816
Indel	Complex regions	F1-score	D-DV	G-D	0.011205724790	0.0096627084626	0.012748741117
Indel	Complex regions	F1-score	D-DV	G-DV	0.014729245798	0.0139917204866	0.015466771110
Indel	Complex regions	F1-score	D-DV	G-G	0.006428170168	0.0050718808783	0.007784459458
Indel	Complex regions	F1-score	D-G	G-D	0.015080514706	0.0145112224965	0.015649806915

Type	Region	Metric	Pipeline 1	Pipeline 2	Mean difference	CI low	CI high
Indel	Complex regions	F1-score	D-G	G-DV	0.018604035714	0.0168715821193	0.020336489309
Indel	Complex regions	F1-score	D-G	G-G	0.010302960084	0.0098960161808	0.010709903987
Indel	Complex regions	F1-score	G-D	G-DV	0.003523521008	0.0015040720122	0.005542970005
Indel	Complex regions	F1-score	G-D	G-G	-0.004777554622	-0.0051838895401	-0.004371219704
Indel	Complex regions	F1-score	G-DV	G-G	-0.008301075630	-0.0101188535565	-0.006483297704
Indel	Simple regions	Precision	D-D	D-DV	-0.000750405882	-0.0009639653545	-0.000536846410
Indel	Simple regions	Precision	D-D	D-G	0.001800670588	0.0013950818981	0.002206259278
Indel	Simple regions	Precision	D-D	G-D	0.000341850000	0.0001117368514	0.000571963149
Indel	Simple regions	Precision	D-D	G-DV	-0.000336494118	-0.0005645441629	-0.000108444072
Indel	Simple regions	Precision	D-D	G-G	-0.000047897059	-0.0003054341506	0.000209640033
Indel	Simple regions	Precision	D-DV	D-G	0.002551076471	0.0020707693441	0.003031383597
Indel	Simple regions	Precision	D-DV	G-D	0.001092255882	0.0008691381161	0.001315373649
Indel	Simple regions	Precision	D-DV	G-DV	0.000413911765	0.0002810286459	0.000546794883
Indel	Simple regions	Precision	D-DV	G-G	0.000702508824	0.0005250895269	0.000879928120

Type	Region	Metric	Pipeline 1	Pipeline 2	Mean difference	CI low	CI high
Indel	Simple regions	Precision	D-G	G-D	-0.001458820588	-0.0018647552350	-0.001052885941
Indel	Simple regions	Precision	D-G	G-DV	-0.002137164706	-0.0026027803546	-0.001671549057
Indel	Simple regions	Precision	D-G	G-G	-0.001848567647	-0.0022838182309	-0.001413317063
Indel	Simple regions	Precision	G-D	G-DV	-0.000678344118	-0.0009083438012	-0.000448344434
Indel	Simple regions	Precision	G-D	G-G	-0.000389747059	-0.0006157500923	-0.000163744025
Indel	Simple regions	Precision	G-DV	G-G	0.000288597059	0.0000734124555	0.000503781662
Indel	Simple regions	Recall	D-D	D-DV	0.001631776471	0.0012093944731	0.002054158468
Indel	Simple regions	Recall	D-D	D-G	0.000057122059	-0.0000831107574	0.000197354875
Indel	Simple regions	Recall	D-D	G-D	0.011145985294	0.0103932640158	0.011898706572
Indel	Simple regions	Recall	D-D	G-DV	0.012236929412	0.0114573911343	0.013016467689
Indel	Simple regions	Recall	D-D	G-G	0.010357741176	0.0095921816645	0.011123300688
Indel	Simple regions	Recall	D-DV	D-G	-0.001574654412	-0.0019859224198	-0.001163386404
Indel	Simple regions	Recall	D-DV	G-D	0.009514208824	0.0086115091175	0.010416908530
Indel	Simple regions	Recall	D-DV	G-DV	0.010605152941	0.0098405013686	0.011369804514

Type	Region	Metric	Pipeline 1	Pipeline 2	Mean difference	CI low	CI high
Indel	Simple regions	Recall	D-DV	G-G	0.008725964706	0.0077720365435	0.009679892868
Indel	Simple regions	Recall	D-G	G-D	0.011088863235	0.0103360730027	0.011841653468
Indel	Simple regions	Recall	D-G	G-DV	0.012179807353	0.0114031522734	0.012956462432
Indel	Simple regions	Recall	D-G	G-G	0.010300619118	0.0095444255900	0.011056812645
Indel	Simple regions	Recall	G-D	G-DV	0.001090944118	0.0007043430773	0.001477545158
Indel	Simple regions	Recall	G-D	G-G	-0.000788244118	-0.0009760545903	-0.000600433645
Indel	Simple regions	Recall	G-DV	G-G	-0.001879188235	-0.0023686466152	-0.001389729855
Indel	Simple regions	F1-score	D-D	D-DV	0.000643302521	0.0003467799977	0.000939825044
Indel	Simple regions	F1-score	D-D	D-G	0.001338096639	0.0010183040470	0.001657889230
Indel	Simple regions	F1-score	D-D	G-D	0.008278224790	0.0078777350874	0.008678714492
Indel	Simple regions	F1-score	D-D	G-DV	0.008588111345	0.0081697586610	0.009006464028
Indel	Simple regions	F1-score	D-D	G-G	0.007433250000	0.0069538318350	0.007912668165
Indel	Simple regions	F1-score	D-DV	D-G	0.000694794118	0.0004024286862	0.000987159549
Indel	Simple regions	F1-score	D-DV	G-D	0.007634922269	0.0071206690606	0.008149175477

Type	Region	Metric	Pipeline 1	Pipeline 2	Mean difference	CI low	CI high
Indel	Simple regions	F1-score	D-DV	G-DV	0.007944808824	0.0075098567323	0.008379760915
Indel	Simple regions	F1-score	D-DV	G-G	0.006789947479	0.0061814865152	0.007398408443
Indel	Simple regions	F1-score	D-G	G-D	0.006940128151	0.0064343218614	0.007445934441
Indel	Simple regions	F1-score	D-G	G-DV	0.007250014706	0.0067828174703	0.007717211941
Indel	Simple regions	F1-score	D-G	G-G	0.006095153361	0.0055118185424	0.006678488180
Indel	Simple regions	F1-score	G-D	G-DV	0.000309886555	-0.0000003123001	0.000620085409
Indel	Simple regions	F1-score	G-D	G-G	-0.000844974790	-0.0011158756805	-0.000574073899
Indel	Simple regions	F1-score	G-DV	G-G	-0.001154861345	-0.0015593388930	-0.000750383796
Indel	Non-coding regions	Precision	D-D	D-DV	-0.000642501471	-0.0009182099443	-0.000366792997
Indel	Non-coding regions	Precision	D-D	D-G	0.002167710294	0.0018795838515	0.002455836737
Indel	Non-coding regions	Precision	D-D	G-D	0.004016692647	0.0035978696847	0.004435515609
Indel	Non-coding regions	Precision	D-D	G-DV	0.000754539706	0.0004539035425	0.001055175869
Indel	Non-coding regions	Precision	D-D	G-G	0.000884355882	0.0005271710288	0.001241540736
Indel	Non-coding regions	Precision	D-DV	D-G	0.002810211765	0.0023991720090	0.003221251520

Type	Region	Metric	Pipeline 1	Pipeline 2	Mean difference	CI low	CI high
Indel	Non-coding regions	Precision	D-DV	G-D	0.004659194118	0.0043071057623	0.005011282473
Indel	Non-coding regions	Precision	D-DV	G-DV	0.001397041176	0.0011893768389	0.001604705514
Indel	Non-coding regions	Precision	D-DV	G-G	0.001526857353	0.0012835828070	0.001770131899
Indel	Non-coding regions	Precision	D-G	G-D	0.001848982353	0.0013561461480	0.002341818558
Indel	Non-coding regions	Precision	D-G	G-DV	-0.001413170588	-0.0018082146281	-0.001018126548
Indel	Non-coding regions	Precision	D-G	G-G	-0.001283354412	-0.0017288624966	-0.000837846327
Indel	Non-coding regions	Precision	G-D	G-DV	-0.003262152941	-0.0036660417550	-0.002858264127
Indel	Non-coding regions	Precision	G-D	G-G	-0.003132336765	-0.0034072018140	-0.002857471715
Indel	Non-coding regions	Precision	G-DV	G-G	0.000129816176	-0.0002034787731	0.000463111126
Indel	Non-coding regions	Recall	D-D	D-DV	0.008777817647	0.0074565582499	0.010099077044
Indel	Non-coding regions	Recall	D-D	D-G	0.003116401471	0.0028135721186	0.003419230823
Indel	Non-coding regions	Recall	D-D	G-D	0.019201002941	0.0181322565018	0.020269749381
Indel	Non-coding regions	Recall	D-D	G-DV	0.024528923529	0.0225916650485	0.026466182010
Indel	Non-coding regions	Recall	D-D	G-G	0.016975835294	0.0159910467895	0.017960623799

Type	Region	Metric	Pipeline 1	Pipeline 2	Mean difference	CI low	CI high
Indel	Non-coding regions	Recall	D-DV	D-G	-0.005661416176	-0.0069712336630	-0.004351598690
Indel	Non-coding regions	Recall	D-DV	G-D	0.010423185294	0.0089125924827	0.011933778106
Indel	Non-coding regions	Recall	D-DV	G-DV	0.015751105882	0.0148137909113	0.016688420853
Indel	Non-coding regions	Recall	D-DV	G-G	0.008198017647	0.0067955101903	0.009600525104
Indel	Non-coding regions	Recall	D-G	G-D	0.016084601471	0.0152094327629	0.016959770178
Indel	Non-coding regions	Recall	D-G	G-DV	0.021412522059	0.0195699866777	0.023255057440
Indel	Non-coding regions	Recall	D-G	G-G	0.013859433824	0.0130827742674	0.014636093380
Indel	Non-coding regions	Recall	G-D	G-DV	0.005327920588	0.0037330197192	0.006922821457
Indel	Non-coding regions	Recall	G-D	G-G	-0.002225167647	-0.0024738876756	-0.001976447618
Indel	Non-coding regions	Recall	G-DV	G-G	-0.007553088235	-0.0090772838564	-0.006028892614
Indel	Non-coding regions	F1-score	D-D	D-DV	0.006126983193	0.0052749544506	0.006979011936
Indel	Non-coding regions	F1-score	D-D	D-G	0.003768920168	0.0034855131963	0.004052327140
Indel	Non-coding regions	F1-score	D-D	G-D	0.016790661765	0.0163264805956	0.017254842934
Indel	Non-coding regions	F1-score	D-D	G-DV	0.018740487395	0.0175779538188	0.019903020971

Type	Region	Metric	Pipeline 1	Pipeline 2	Mean difference	CI low	CI high
Indel	Non-coding regions	F1-score	D-D	G-G	0.012991714286	0.0125672040818	0.013416224490
Indel	Non-coding regions	F1-score	D-DV	D-G	-0.002358063025	-0.0031713347208	-0.001544791330
Indel	Non-coding regions	F1-score	D-DV	G-D	0.010663678571	0.0095263556489	0.011801001494
Indel	Non-coding regions	F1-score	D-DV	G-DV	0.012613504202	0.0121043447834	0.013122663620
Indel	Non-coding regions	F1-score	D-DV	G-G	0.006864731092	0.0058161106067	0.007913351578
Indel	Non-coding regions	F1-score	D-G	G-D	0.013021741597	0.0125420936754	0.013501389518
Indel	Non-coding regions	F1-score	D-G	G-DV	0.014971567227	0.0138719088099	0.016071225644
Indel	Non-coding regions	F1-score	D-G	G-G	0.009222794118	0.0088350600797	0.009610528156
Indel	Non-coding regions	F1-score	G-D	G-DV	0.001949825630	0.0005763781721	0.003323273088
Indel	Non-coding regions	F1-score	G-D	G-G	-0.003798947479	-0.0040875328205	-0.003510362137
Indel	Non-coding regions	F1-score	G-DV	G-G	-0.005748773109	-0.0070212402479	-0.004476305971
Indel	Coding regions	Precision	D-D	D-DV	0.010514602941	-0.0035967486692	0.024625954552
Indel	Coding regions	Precision	D-D	D-G	0.046653198529	0.0319321887688	0.061374208290
Indel	Coding regions	Precision	D-D	G-D	0.009548119118	-0.0047035661939	0.023799804429

Type	Region	Metric	Pipeline 1	Pipeline 2	Mean difference	CI low	CI high
Indel	Coding regions	Precision	D-D	G-DV	-0.000407822059	-0.0133470765661	0.012531432448
Indel	Coding regions	Precision	D-D	G-G	0.004527338235	-0.0094506111176	0.018505287588
Indel	Coding regions	Precision	D-DV	D-G	0.036138595588	0.0209121441684	0.051365047008
Indel	Coding regions	Precision	D-DV	G-D	-0.000966483824	-0.0174958494858	0.015562881839
Indel	Coding regions	Precision	D-DV	G-DV	-0.010922425000	-0.0217521642935	-0.000092685707
Indel	Coding regions	Precision	D-DV	G-G	-0.005987264706	-0.0222288670301	0.010254337618
Indel	Coding regions	Precision	D-G	G-D	-0.037105079412	-0.0528423460635	-0.021367812760
Indel	Coding regions	Precision	D-G	G-DV	-0.047061020588	-0.0632211424458	-0.030900898731
Indel	Coding regions	Precision	D-G	G-G	-0.042125860294	-0.0575200161153	-0.026731704473
Indel	Coding regions	Precision	G-D	G-DV	-0.009955941176	-0.0249102210481	0.004998338695
Indel	Coding regions	Precision	G-D	G-G	-0.005020780882	-0.0079782648203	-0.002063296944
Indel	Coding regions	Precision	G-DV	G-G	0.004935160294	-0.0097216786718	0.019591999260
Indel	Coding regions	Recall	D-D	D-DV	0.018273779412	0.0043706390638	0.032176919760
Indel	Coding regions	Recall	D-D	D-G	0.038245701471	0.0239100562862	0.052581346655

Type	Region	Metric	Pipeline 1	Pipeline 2	Mean difference	CI low	CI high
Indel	Coding regions	Recall	D-D	G-D	0.043830920588	0.0293763506969	0.058285490480
Indel	Coding regions	Recall	D-D	G-DV	0.038997227941	0.0256119034651	0.052382552417
Indel	Coding regions	Recall	D-D	G-G	0.037078047059	0.0229013382889	0.051254755829
Indel	Coding regions	Recall	D-DV	D-G	0.019971922059	0.0051717969695	0.034772047148
Indel	Coding regions	Recall	D-DV	G-D	0.025557141176	0.0086248268067	0.042489455546
Indel	Coding regions	Recall	D-DV	G-DV	0.020723448529	0.0089631158371	0.032483781222
Indel	Coding regions	Recall	D-DV	G-G	0.018804267647	0.0021244334563	0.035484101838
Indel	Coding regions	Recall	D-G	G-D	0.005585219118	-0.0105926730399	0.021763111275
Indel	Coding regions	Recall	D-G	G-DV	0.000751526471	-0.0159791431091	0.017482196050
Indel	Coding regions	Recall	D-G	G-G	-0.001167654412	-0.0170042155574	0.014668906734
Indel	Coding regions	Recall	G-D	G-DV	-0.004833692647	-0.0195905979482	0.009923212654
Indel	Coding regions	Recall	G-D	G-G	-0.006752873529	-0.0097243302126	-0.003781416846
Indel	Coding regions	Recall	G-DV	G-G	-0.001919180882	-0.0164302123399	0.012591850575
Indel	Coding regions	F1-score	D-D	D-DV	-0.002933827273	-0.0060777900129	0.000210135467

Type	Region	Metric	Pipeline 1	Pipeline 2	Mean difference	CI low	CI high
Indel	Coding regions	F1-score	D-D	D-G	0.009759881944	0.0047814165768	0.014738347312
Indel	Coding regions	F1-score	D-D	G-D	0.034668132883	0.0283075284193	0.041028737347
Indel	Coding regions	F1-score	D-D	G-DV	0.025394690583	0.0205547405170	0.030234640649
Indel	Coding regions	F1-score	D-D	G-G	0.025248871622	0.0204085046879	0.030089238555
Indel	Coding regions	F1-score	D-DV	D-G	0.012927589202	0.0079677570949	0.017887421309
Indel	Coding regions	F1-score	D-DV	G-D	0.038441324885	0.0329008854491	0.043981764320
Indel	Coding regions	F1-score	D-DV	G-DV	0.028480623025	0.0251448343902	0.031816411659
Indel	Coding regions	F1-score	D-DV	G-G	0.028805029954	0.0251528209386	0.032457238969
Indel	Coding regions	F1-score	D-G	G-D	0.026052172494	0.0184643824931	0.033639962495
Indel	Coding regions	F1-score	D-G	G-DV	0.016611595794	0.0101528716261	0.023070319963
Indel	Coding regions	F1-score	D-G	G-G	0.016303566434	0.0101664724586	0.022440660409
Indel	Coding regions	F1-score	G-D	G-DV	-0.009221358277	-0.0142758435588	-0.004166872994
Indel	Coding regions	F1-score	G-D	G-G	-0.009232123620	-0.0135311850625	-0.004933062178
Indel	Coding regions	F1-score	G-DV	G-G	-0.000261979592	-0.0027825279331	0.002258568749

Type	Region	Metric	Pipeline 1	Pipeline 2	Mean difference	CI low	CI high
SNV	All regions	Precision	D-D	D-DV	-0.001023691176	-0.0010523507074	-0.000995031646
SNV	All regions	Precision	D-D	D-G	0.000682705882	0.0005866871382	0.000778724627
SNV	All regions	Precision	D-D	G-D	0.003464735294	0.0033908249456	0.003538645643
SNV	All regions	Precision	D-D	G-DV	-0.000938955882	-0.0009800944136	-0.000897817351
SNV	All regions	Precision	D-D	G-G	0.005518705882	0.0054470384945	0.005590373270
SNV	All regions	Precision	D-DV	D-G	0.001706397059	0.0016141830487	0.001798611069
SNV	All regions	Precision	D-DV	G-D	0.004488426471	0.0044037867167	0.004573066224
SNV	All regions	Precision	D-DV	G-DV	0.000084735294	0.0000439867302	0.000125483858
SNV	All regions	Precision	D-DV	G-G	0.006542397059	0.0064679922326	0.006616801885
SNV	All regions	Precision	D-G	G-D	0.002782029412	0.0026498606484	0.002914198175
SNV	All regions	Precision	D-G	G-DV	-0.001621661765	-0.0017275792262	-0.001515744303
SNV	All regions	Precision	D-G	G-G	0.004836000000	0.0047560401744	0.004915959826
SNV	All regions	Precision	G-D	G-DV	-0.004403691176	-0.0044635424505	-0.004343839902
SNV	All regions	Precision	G-D	G-G	0.002053970588	0.0019441121978	0.002163828979

Type	Region	Metric	Pipeline 1	Pipeline 2	Mean difference	CI low	CI high
SNV	All regions	Precision	G-DV	G-G	0.006457661765	0.0063736725977	0.006541650932
SNV	All regions	Recall	D-D	D-DV	0.000597970588	0.0005719971839	0.000623943993
SNV	All regions	Recall	D-D	D-G	0.003345470588	0.0033085916853	0.003382349491
SNV	All regions	Recall	D-D	G-D	0.023620161765	0.0235645544426	0.023675769087
SNV	All regions	Recall	D-D	G-DV	0.021002470588	0.0209690979722	0.021035843204
SNV	All regions	Recall	D-D	G-G	0.023684161765	0.0236339198538	0.023734403676
SNV	All regions	Recall	D-DV	D-G	0.002747500000	0.0027011980303	0.002793801970
SNV	All regions	Recall	D-DV	G-D	0.023022191176	0.0229579093579	0.023086472995
SNV	All regions	Recall	D-DV	G-DV	0.020404500000	0.0203617307622	0.020447269238
SNV	All regions	Recall	D-DV	G-G	0.023086191176	0.0230220090541	0.023150373299
SNV	All regions	Recall	D-G	G-D	0.020274691176	0.0202286387075	0.020320743645
SNV	All regions	Recall	D-G	G-DV	0.017657000000	0.0176206397260	0.017693360274
SNV	All regions	Recall	D-G	G-G	0.020338691176	0.0202942032873	0.020383179066
SNV	All regions	Recall	G-D	G-DV	-0.002617691176	-0.0026586678786	-0.002576714474

Type	Region	Metric	Pipeline 1	Pipeline 2	Mean difference	CI low	CI high
SNV	All regions	Recall	G-D	G-G	0.000064000000	0.0000225705694	0.000105429431
SNV	All regions	Recall	G-DV	G-G	0.002681691176	0.0026387330304	0.002724649323
SNV	All regions	F1-score	D-D	D-DV	-0.000211602941	-0.0002348969903	-0.000188308892
SNV	All regions	F1-score	D-D	D-G	0.002016941176	0.0019688279649	0.002065054388
SNV	All regions	F1-score	D-D	G-D	0.013653426471	0.0135970088167	0.013709844124
SNV	All regions	F1-score	D-D	G-DV	0.010162073529	0.0101304872411	0.010193659818
SNV	All regions	F1-score	D-D	G-G	0.014692367647	0.0146528982749	0.014731837019
SNV	All regions	F1-score	D-DV	D-G	0.002228544118	0.0021810995880	0.002275988647
SNV	All regions	F1-score	D-DV	G-D	0.013865029412	0.0137980552688	0.013932003555
SNV	All regions	F1-score	D-DV	G-DV	0.010373676471	0.0103366161221	0.010410736819
SNV	All regions	F1-score	D-DV	G-G	0.014903970588	0.0148592202238	0.014948720953
SNV	All regions	F1-score	D-G	G-D	0.011636485294	0.0115642126063	0.011708757982
SNV	All regions	F1-score	D-G	G-DV	0.008145132353	0.0080894848773	0.008200779829
SNV	All regions	F1-score	D-G	G-G	0.012675426471	0.0126314220566	0.012719430885

Type	Region	Metric	Pipeline 1	Pipeline 2	Mean difference	CI low	CI high
SNV	All regions	F1-score	G-D	G-DV	-0.003491352941	-0.0035339503582	-0.003448755524
SNV	All regions	F1-score	G-D	G-G	0.001038941176	0.0009827237389	0.001095158614
SNV	All regions	F1-score	G-DV	G-G	0.004530294118	0.0044878783694	0.004572709866
SNV	Complex regions	Precision	D-D	D-DV	-0.003816397059	-0.0039212655816	-0.003711528536
SNV	Complex regions	Precision	D-D	D-G	0.000053852941	-0.0001679960229	0.000275701905
SNV	Complex regions	Precision	D-D	G-D	0.013088955882	0.0128257051375	0.013352206627
SNV	Complex regions	Precision	D-D	G-DV	-0.003788705882	-0.0039479601816	-0.003629451583
SNV	Complex regions	Precision	D-D	G-G	0.020281544118	0.0200499413800	0.020513146855
SNV	Complex regions	Precision	D-DV	D-G	0.003870250000	0.0036699946944	0.004070505306
SNV	Complex regions	Precision	D-DV	G-D	0.016905352941	0.0166020228753	0.017208683007
SNV	Complex regions	Precision	D-DV	G-DV	0.000027691176	-0.0001341196468	0.000189502000
SNV	Complex regions	Precision	D-DV	G-G	0.024097941176	0.0238618583419	0.024334024011
SNV	Complex regions	Precision	D-G	G-D	0.013035102941	0.0126486117665	0.013421594116
SNV	Complex regions	Precision	D-G	G-DV	-0.003842558824	-0.0041192679096	-0.003565849737

Type	Region	Metric	Pipeline 1	Pipeline 2	Mean difference	CI low	CI high
SNV	Complex regions	Precision	D-G	G-G	0.020227691176	0.0199746138692	0.020480768484
SNV	Complex regions	Precision	G-D	G-DV	-0.016877661765	-0.0170807186178	-0.016674604912
SNV	Complex regions	Precision	G-D	G-G	0.007192588235	0.0068216707169	0.007563505754
SNV	Complex regions	Precision	G-DV	G-G	0.024070250000	0.0237915647982	0.024348935202
SNV	Complex regions	Recall	D-D	D-DV	0.002298029412	0.0021950839549	0.002400974869
SNV	Complex regions	Recall	D-D	D-G	0.012671176471	0.0125283062778	0.012814046663
SNV	Complex regions	Recall	D-D	G-D	0.047312838235	0.0471228952347	0.047502781236
SNV	Complex regions	Recall	D-D	G-DV	0.037938352941	0.0378179311002	0.038058774782
SNV	Complex regions	Recall	D-D	G-G	0.047766676471	0.0475781209391	0.047955232002
SNV	Complex regions	Recall	D-DV	D-G	0.010373147059	0.0101942169504	0.010552077167
SNV	Complex regions	Recall	D-DV	G-D	0.045014808824	0.0447885000993	0.045241117548
SNV	Complex regions	Recall	D-DV	G-DV	0.035640323529	0.0354844289896	0.035796218069
SNV	Complex regions	Recall	D-DV	G-G	0.045468647059	0.0452278877949	0.045709406323
SNV	Complex regions	Recall	D-G	G-D	0.034641661765	0.0344801630264	0.034803160503

Type	Region	Metric	Pipeline 1	Pipeline 2	Mean difference	CI low	CI high
SNV	Complex regions	Recall	D-G	G-DV	0.025267176471	0.0251310533696	0.025403299572
SNV	Complex regions	Recall	D-G	G-G	0.035095500000	0.0349243367704	0.035266663230
SNV	Complex regions	Recall	G-D	G-DV	-0.009374485294	-0.0095107569124	-0.009238213676
SNV	Complex regions	Recall	G-D	G-G	0.000453838235	0.0003059622597	0.000601714211
SNV	Complex regions	Recall	G-DV	G-G	0.009828323529	0.0096637073339	0.009992939725
SNV	Complex regions	F1-score	D-D	D-DV	-0.000740411765	-0.0008291988914	-0.000651624638
SNV	Complex regions	F1-score	D-D	D-G	0.006422661765	0.0063029292572	0.006542394272
SNV	Complex regions	F1-score	D-D	G-D	0.030560735294	0.0303672118932	0.030754258695
SNV	Complex regions	F1-score	D-D	G-DV	0.017587897059	0.0174680695379	0.017707724580
SNV	Complex regions	F1-score	D-D	G-G	0.034265823529	0.0341311848079	0.034400462251
SNV	Complex regions	F1-score	D-DV	D-G	0.007163073529	0.0070390617951	0.007287085264
SNV	Complex regions	F1-score	D-DV	G-D	0.031301147059	0.0310651212869	0.031537172831
SNV	Complex regions	F1-score	D-DV	G-DV	0.018328308824	0.0181871267567	0.018469490890
SNV	Complex regions	F1-score	D-DV	G-G	0.035006235294	0.0348538397208	0.035158630867

Type	Region	Metric	Pipeline 1	Pipeline 2	Mean difference	CI low	CI high
SNV	Complex regions	F1-score	D-G	G-D	0.024138073529	0.0239239711421	0.024352175917
SNV	Complex regions	F1-score	D-G	G-DV	0.011165235294	0.0110172696943	0.011313200894
SNV	Complex regions	F1-score	D-G	G-G	0.027843161765	0.0276974319796	0.027988891550
SNV	Complex regions	F1-score	G-D	G-DV	-0.012972838235	-0.0131087000484	-0.012836976422
SNV	Complex regions	F1-score	G-D	G-G	0.003705088235	0.0035262470981	0.003883929372
SNV	Complex regions	F1-score	G-DV	G-G	0.016677926471	0.0165425071235	0.016813345818
SNV	Simple regions	Precision	D-D	D-DV	-0.000038000000	-0.0000446021540	-0.000031397846
SNV	Simple regions	Precision	D-D	D-G	0.000935102941	0.0008640249127	0.001006180970
SNV	Simple regions	Precision	D-D	G-D	0.000217647059	0.0001940383847	0.000241255733
SNV	Simple regions	Precision	D-D	G-DV	0.000100588235	0.0000922113327	0.000108965138
SNV	Simple regions	Precision	D-D	G-G	0.000459808824	0.0004134860695	0.000506131578
SNV	Simple regions	Precision	D-DV	D-G	0.000973102941	0.0009016053078	0.001044600575
SNV	Simple regions	Precision	D-DV	G-D	0.000255647059	0.0002316564084	0.000279637709
SNV	Simple regions	Precision	D-DV	G-DV	0.000138588235	0.0001323619149	0.000144814556

Type	Region	Metric	Pipeline 1	Pipeline 2	Mean difference	CI low	CI high
SNV	Simple regions	Precision	D-DV	G-G	0.000497808824	0.0004503504020	0.000545267245
SNV	Simple regions	Precision	D-G	G-D	-0.000717455882	-0.0007880304211	-0.000646881344
SNV	Simple regions	Precision	D-G	G-DV	-0.000834514706	-0.0009044585950	-0.000764570817
SNV	Simple regions	Precision	D-G	G-G	-0.000475294118	-0.0005189978872	-0.000431590348
SNV	Simple regions	Precision	G-D	G-DV	-0.000117058824	-0.0001408153466	-0.000093302300
SNV	Simple regions	Precision	G-D	G-G	0.000242161765	0.0001948567218	0.000289466808
SNV	Simple regions	Precision	G-DV	G-G	0.000359220588	0.0003134380406	0.000405003136
SNV	Simple regions	Recall	D-D	D-DV	0.000001926471	-0.0000020049155	0.000005857857
SNV	Simple regions	Recall	D-D	D-G	0.000076661765	0.0000699551895	0.000083368340
SNV	Simple regions	Recall	D-D	G-D	0.015315470588	0.0152944034676	0.015336537709
SNV	Simple regions	Recall	D-D	G-DV	0.015066073529	0.0150560672082	0.015076079851
SNV	Simple regions	Recall	D-D	G-G	0.015242852941	0.0152308697182	0.015254836164
SNV	Simple regions	Recall	D-DV	D-G	0.000074735294	0.0000681829150	0.000081287673
SNV	Simple regions	Recall	D-DV	G-D	0.015313544118	0.0152919383402	0.015335149895

Type	Region	Metric	Pipeline 1	Pipeline 2	Mean difference	CI low	CI high
SNV	Simple regions	Recall	D-DV	G-DV	0.015064147059	0.0150551615400	0.015073132578
SNV	Simple regions	Recall	D-DV	G-G	0.015240926471	0.0152294619294	0.015252391012
SNV	Simple regions	Recall	D-G	G-D	0.015238808824	0.0152169762962	0.015260641351
SNV	Simple regions	Recall	D-G	G-DV	0.014989411765	0.0149779006438	0.015000922886
SNV	Simple regions	Recall	D-G	G-G	0.015166191176	0.0151538136681	0.015178568685
SNV	Simple regions	Recall	G-D	G-DV	-0.000249397059	-0.0002721275324	-0.000226666585
SNV	Simple regions	Recall	G-D	G-G	-0.000072617647	-0.0000939111238	-0.000051324170
SNV	Simple regions	Recall	G-DV	G-G	0.000176779412	0.0001656125064	0.000187946317
SNV	Simple regions	F1-score	D-D	D-DV	-0.000018058824	-0.0000219273295	-0.000014190318
SNV	Simple regions	F1-score	D-D	D-G	0.000506029412	0.0004706025443	0.000541456279
SNV	Simple regions	F1-score	D-D	G-D	0.007823691176	0.0078033108500	0.007844071503
SNV	Simple regions	F1-score	D-D	G-DV	0.007639411765	0.0076331522471	0.007645671282
SNV	Simple regions	F1-score	D-D	G-G	0.007906058824	0.0078809144525	0.007931203195
SNV	Simple regions	F1-score	D-DV	D-G	0.000524088235	0.0004885338163	0.000559642654

Type	Region	Metric	Pipeline 1	Pipeline 2	Mean difference	CI low	CI high
SNV	Simple regions	F1-score	D-DV	G-D	0.007841750000	0.0078209808204	0.007862519180
SNV	Simple regions	F1-score	D-DV	G-DV	0.007657470588	0.0076522076484	0.007662733528
SNV	Simple regions	F1-score	D-DV	G-G	0.007924117647	0.0078986764052	0.007949558889
SNV	Simple regions	F1-score	D-G	G-D	0.007317661765	0.0072777039468	0.007357619583
SNV	Simple regions	F1-score	D-G	G-DV	0.007133382353	0.0070971499119	0.007169614794
SNV	Simple regions	F1-score	D-G	G-G	0.007400029412	0.0073765491632	0.007423509660
SNV	Simple regions	F1-score	G-D	G-DV	-0.000184279412	-0.0002061657279	-0.000162393096
SNV	Simple regions	F1-score	G-D	G-G	0.000082367647	0.0000516753413	0.000113059953
SNV	Simple regions	F1-score	G-DV	G-G	0.000266647059	0.0002413222176	0.000291971900
SNV	Non-coding regions	Precision	D-D	D-DV	-0.001028794118	-0.0010575102324	-0.001000078003
SNV	Non-coding regions	Precision	D-D	D-G	0.000671588235	0.0005762799662	0.000766896504
SNV	Non-coding regions	Precision	D-D	G-D	0.003468102941	0.0033944757215	0.003541730161
SNV	Non-coding regions	Precision	D-D	G-DV	-0.000954338235	-0.0009952418313	-0.000913434639
SNV	Non-coding regions	Precision	D-D	G-G	0.005498088235	0.0054257831978	0.005570393273

Type	Region	Metric	Pipeline 1	Pipeline 2	Mean difference	CI low	CI high
SNV	Non-coding regions	Precision	D-DV	D-G	0.001700382353	0.0016089755581	0.001791789148
SNV	Non-coding regions	Precision	D-DV	G-D	0.004496897059	0.0044119607624	0.004581833355
SNV	Non-coding regions	Precision	D-DV	G-DV	0.000074455882	0.0000337073438	0.000115204421
SNV	Non-coding regions	Precision	D-DV	G-G	0.006526882353	0.0064517006190	0.006602064087
SNV	Non-coding regions	Precision	D-G	G-D	0.002796514706	0.0026645100513	0.002928519360
SNV	Non-coding regions	Precision	D-G	G-DV	-0.001625926471	-0.0017314536478	-0.001520399293
SNV	Non-coding regions	Precision	D-G	G-G	0.004826500000	0.0047446245739	0.004908375426
SNV	Non-coding regions	Precision	G-D	G-DV	-0.004422441176	-0.0044831160820	-0.004361766271
SNV	Non-coding regions	Precision	G-D	G-G	0.002029985294	0.0019189222460	0.002141048342
SNV	Non-coding regions	Precision	G-DV	G-G	0.006452426471	0.0063679483640	0.006536904577
SNV	Non-coding regions	Recall	D-D	D-DV	0.000597852941	0.0005714514364	0.000624254446
SNV	Non-coding regions	Recall	D-D	D-G	0.003328426471	0.0032914371688	0.003365415772
SNV	Non-coding regions	Recall	D-D	G-D	0.023471691176	0.0234158468446	0.023527535508
SNV	Non-coding regions	Recall	D-D	G-DV	0.020846779412	0.0208133992141	0.020880159609

Type	Region	Metric	Pipeline 1	Pipeline 2	Mean difference	CI low	CI high
SNV	Non-coding regions	Recall	D-D	G-G	0.023543455882	0.0234937734062	0.023593138359
SNV	Non-coding regions	Recall	D-DV	D-G	0.002730573529	0.0026846139112	0.002776533148
SNV	Non-coding regions	Recall	D-DV	G-D	0.022873838235	0.0228100096756	0.022937666795
SNV	Non-coding regions	Recall	D-DV	G-DV	0.020248926471	0.0202068863004	0.020290966641
SNV	Non-coding regions	Recall	D-DV	G-G	0.022945602941	0.0228822335429	0.023008972339
SNV	Non-coding regions	Recall	D-G	G-D	0.020143264706	0.0200961932134	0.020190336198
SNV	Non-coding regions	Recall	D-G	G-DV	0.017518352941	0.0174816686488	0.017555037234
SNV	Non-coding regions	Recall	D-G	G-G	0.020215029412	0.0201700718658	0.020259986958
SNV	Non-coding regions	Recall	G-D	G-DV	-0.002624911765	-0.0026660927510	-0.002583730778
SNV	Non-coding regions	Recall	G-D	G-G	0.000071764706	0.0000302916249	0.000113237787
SNV	Non-coding regions	Recall	G-DV	G-G	0.002696676471	0.0026536754518	0.002739677489
SNV	Non-coding regions	F1-score	D-D	D-DV	-0.000214147059	-0.0002376200177	-0.000190674100
SNV	Non-coding regions	F1-score	D-D	D-G	0.002002808824	0.0019547778815	0.002050839766
SNV	Non-coding regions	F1-score	D-D	G-D	0.013579147059	0.0135228680047	0.013635426113

Type	Region	Metric	Pipeline 1	Pipeline 2	Mean difference	CI low	CI high
SNV	Non-coding regions	F1-score	D-D	G-DV	0.010074735294	0.0100433335858	0.010106137002
SNV	Non-coding regions	F1-score	D-D	G-G	0.014610455882	0.0145711240132	0.014649787751
SNV	Non-coding regions	F1-score	D-DV	D-G	0.002216955882	0.0021699023982	0.002264009367
SNV	Non-coding regions	F1-score	D-DV	G-D	0.013793294118	0.0137265616454	0.013860026590
SNV	Non-coding regions	F1-score	D-DV	G-DV	0.010288882353	0.0102523604492	0.010325404257
SNV	Non-coding regions	F1-score	D-DV	G-G	0.014824602941	0.0147803167911	0.014868889091
SNV	Non-coding regions	F1-score	D-G	G-D	0.011576338235	0.0115038050057	0.011648871465
SNV	Non-coding regions	F1-score	D-G	G-DV	0.008071926471	0.0080162824772	0.008127570464
SNV	Non-coding regions	F1-score	D-G	G-G	0.012607647059	0.0125628023177	0.012652491800
SNV	Non-coding regions	F1-score	G-D	G-DV	-0.003504411765	-0.0035474951826	-0.003461328347
SNV	Non-coding regions	F1-score	G-D	G-G	0.001031308824	0.0009744738014	0.001088143846
SNV	Non-coding regions	F1-score	G-DV	G-G	0.004535720588	0.0044933822266	0.004578058950
SNV	Coding regions	Precision	D-D	D-DV	-0.000403426471	-0.0005908477869	-0.000216005154
SNV	Coding regions	Precision	D-D	D-G	0.002035352941	0.0014632074433	0.002607498439

Type	Region	Metric	Pipeline 1	Pipeline 2	Mean difference	CI low	CI high
SNV	Coding regions	Precision	D-D	G-D	0.003023588235	0.0025966609456	0.003450515525
SNV	Coding regions	Precision	D-D	G-DV	0.000976088235	0.0006429392766	0.001309237194
SNV	Coding regions	Precision	D-D	G-G	0.008070808824	0.0074478875455	0.008693730102
SNV	Coding regions	Precision	D-DV	D-G	0.002438779412	0.0018568994181	0.003020659405
SNV	Coding regions	Precision	D-DV	G-D	0.003427014706	0.0030608909021	0.003793138510
SNV	Coding regions	Precision	D-DV	G-DV	0.001379514706	0.0011020869292	0.001656942483
SNV	Coding regions	Precision	D-DV	G-G	0.008474235294	0.0078691260707	0.009079344518
SNV	Coding regions	Precision	D-G	G-D	0.000988235294	0.0003019611143	0.001674509474
SNV	Coding regions	Precision	D-G	G-DV	-0.001059264706	-0.0017160139615	-0.000402515450
SNV	Coding regions	Precision	D-G	G-G	0.006035455882	0.0052041788069	0.006866732958
SNV	Coding regions	Precision	G-D	G-DV	-0.002047500000	-0.0024565094293	-0.001638490571
SNV	Coding regions	Precision	G-D	G-G	0.005047220588	0.0044288615675	0.005665579609
SNV	Coding regions	Precision	G-DV	G-G	0.007094720588	0.0065197895783	0.007669651598
SNV	Coding regions	Recall	D-D	D-DV	0.000610279412	0.0003425576763	0.000878001147

Type	Region	Metric	Pipeline 1	Pipeline 2	Mean difference	CI low	CI high
SNV	Coding regions	Recall	D-D	D-G	0.005423485294	0.0050049942828	0.005841976305
SNV	Coding regions	Recall	D-D	G-D	0.041779367647	0.0413786347031	0.042180100591
SNV	Coding regions	Recall	D-D	G-DV	0.040040632353	0.0397020650056	0.040379199700
SNV	Coding regions	Recall	D-D	G-G	0.040892735294	0.0404560422657	0.041329428323
SNV	Coding regions	Recall	D-DV	D-G	0.004813205882	0.0043579528749	0.005268458890
SNV	Coding regions	Recall	D-DV	G-D	0.041169088235	0.0407281570917	0.041610019379
SNV	Coding regions	Recall	D-DV	G-DV	0.039430352941	0.0391016515989	0.039759054283
SNV	Coding regions	Recall	D-DV	G-G	0.040282455882	0.0398270866857	0.040737825079
SNV	Coding regions	Recall	D-G	G-D	0.036355882353	0.0358855194146	0.036826245291
SNV	Coding regions	Recall	D-G	G-DV	0.034617147059	0.0341650972663	0.035069196851
SNV	Coding regions	Recall	D-G	G-G	0.035469250000	0.0349812682165	0.035957231783
SNV	Coding regions	Recall	G-D	G-DV	-0.001738735294	-0.0021001957273	-0.001377274861
SNV	Coding regions	Recall	G-D	G-G	-0.000886632353	-0.0012005650581	-0.000572699648
SNV	Coding regions	Recall	G-DV	G-G	0.000852102941	0.0004748342821	0.001229371600

Type	Region	Metric	Pipeline 1	Pipeline 2	Mean difference	CI low	CI high
SNV	Coding regions	F1-score	D-D	D-DV	0.000105455882	-0.0000880498174	0.000298961582
SNV	Coding regions	F1-score	D-D	D-G	0.003740220588	0.0033777642789	0.004102676898
SNV	Coding regions	F1-score	D-D	G-D	0.022855676471	0.0225262601835	0.023185092758
SNV	Coding regions	F1-score	D-D	G-DV	0.020967735294	0.0207045421115	0.021230928477
SNV	Coding regions	F1-score	D-D	G-G	0.024817779412	0.0243913968717	0.025244161952
SNV	Coding regions	F1-score	D-DV	D-G	0.003634764706	0.0032620410930	0.004007488319
SNV	Coding regions	F1-score	D-DV	G-D	0.022750220588	0.0224445830715	0.023055858105
SNV	Coding regions	F1-score	D-DV	G-DV	0.020862279412	0.0206137121479	0.021110846676
SNV	Coding regions	F1-score	D-DV	G-G	0.024712323529	0.0242980148511	0.025126632208
SNV	Coding regions	F1-score	D-G	G-D	0.019115455882	0.0187056505661	0.019525261199
SNV	Coding regions	F1-score	D-G	G-DV	0.017227514706	0.0168577374714	0.017597291940
SNV	Coding regions	F1-score	D-G	G-G	0.021077558824	0.0205778145329	0.021577303114
SNV	Coding regions	F1-score	G-D	G-DV	-0.001887941176	-0.0021789706216	-0.001596911731
SNV	Coding regions	F1-score	G-D	G-G	0.001962102941	0.0015829344020	0.002341271480

Type	Region	Metric	Pipeline 1	Pipeline 2	Mean difference	CI low	CI high
SNV	Coding regions	F1-score	G-DV	G-G	0.003850044118	0.0034730736802	0.004227014555
